# Self-harm and suicide in adults: will safety plans keep people safe after self-harm?

**DOI:** 10.1192/bjb.2020.150

**Published:** 2022-02

**Authors:** Allan House

**Affiliations:** School of Medicine, University of Leeds, UK

**Keywords:** Autonomy, consent and capacity, emergency psychiatry, self-harm, suicide prevention

## Abstract

Safety planning is recommended as a part of the response to everybody who presents after self-harm, although there is surprisingly little evidence for its effectiveness. There is potential for such plans to be experienced as unhelpful if patients are not genuinely involved in their production and if the plan does not include information about meaningful sources of support. Staff training is needed to ensure that plans are delivered in a collaborative way and self-harm services need to be improved nationally if such plans are to be effective.

It has been known for a long time that the risk of suicide is greatly increased after hospital presentation due to self-harm.^1^ England's National Suicide Prevention Strategy recognises this risk and its 2017 report ‘expanded the scope of the National Strategy to include self-harm as a new key area for action’.^2^ This welcome policy change brings with it a dilemma – how to respond to self-harm as a suicide risk while at the same time responding to its many other meanings. Suicide is perhaps the worst outcome in psychiatry and inevitably captures our attention – can we develop our services to respond to the risk without at the same time allowing the ‘risk-reduction’ aspect of service provision to overshadow the rest of the process of care? The recent Royal College of Psychiatrists (RCPsych) report *Self-harm and Suicide in Adults* (CR229)^3^ can be read as an attempt to answer this question.

## What is safety planning?

The RCPsych's report is forthright in stating that there is more to assessment after self-harm than judging suicide risk and that management should involve a ‘holistic psychosocial approach’ at the core of which, at least in the short term, is making a safety plan. According to the report, such a plan comprises: individual strategies or activities to instil hope; calming or distracting activities; restriction of access to common means of suicide; and contacts for social and crisis support.^3^ It has to be said that some of the illustrative activities seem a bit weak for the work they have to do – looking at a photo of a great view or doing Sudoku for example – but if the plan is genuinely co-produced then its elements might be expected to make sense to the person to whom it applies.

There are a number of similar approaches to that proposed by the RCPsych, and something very similar forms a part of the risk management plan endorsed by the National Institute for Health and Care Excellence (NICE) as an essential component of the response to self-harm.^4^ On the face of it, it seems like an uncontroversial recommendation that everybody should indeed have something like a safety plan: does that initial impression hold up to closer scrutiny, especially in relation to the dilemma under discussion here?

## How effective is safety planning after self-harm?

The first thing to say is that, given how roundly they are endorsed by the RCPsych, there is surprisingly little evidence to support the use of safety plans as a means of reducing repetition of self-harm. In fact, CR229 cites only one supportive study,^5^ a retrospective case-note review of 48 cases from the USA. Elsewhere, a small randomised controlled trial (*n* = 97) of active-duty US Army soldiers^6^ reported, in a comparison of a crisis response plan versus a ‘contract for safety’, an effect on self-reported attempted suicide at 6 months (3/64 *v.* 5/33 participants). A recent review of suicide prevention interventions^7^ reported a positive effect on subsequent self-harm rates but the positive outcome for that part of the review is accounted for by only two studies: one^8^ is a study of men in active military service which used multiple outcomes and reported a reduction in self-reported attempted suicide but not in emergency department attendances for self-harm. The other^9^ is a non-randomised comparison of Veterans Affairs hospitals; 90% of participants were men with ‘suicide-related concerns’ and the primary outcome (a composite measure of ‘suicidal behaviour') was found in 3–5% at 6 months. The results of these studies are not only unconvincing but they are not generalisable to the UK self-harm population.

Of closer relevance to the position of UK clinicians seeing people after an episode of self-harm is the non-randomised Emergency Department Safety Assessment and Follow-up Evaluation (ED-SAFE) study,^10^ which reported self-reported suicide attempts but not all self-harm episodes, citing a 12-month difference of 20.9% (treatment as usual) *v*. 18.3% (intervention) – a result that is by no means definitive given the study design. The only UK randomised controlled trial of a comparable intervention^11^ – called a volitional help sheet, with many of the features of a suicide prevention plan – found no difference in 6-month self-harm repetition rates between usual care and the new intervention.

## Could safety planning have detrimental effects?

Does the lack of evidence of effectiveness matter? Isn't such a common-sense action worth implementing regardless of limited evidence of its effectiveness? The reason to be cautious is that there are non-trivial possibilities of unwanted outcomes from a misapplication of the approach – misapplication, that is, in the way the plan is introduced, how it is negotiated and what are its specific components. If adverse consequences are to be avoided then they need to be considered by the service during the planning and delivery of suicide prevention plans after self-harm.

One possible problem arises from the degree to which the implementation of the suicide prevention plan is left to the person who has self-harmed. The first four of the six suggestions under the heading ‘sources of support’ in the RCPsych report relate to marshalling social or informal supports,^3^ yet we know that people who self-harm find it difficult to confide in others^12,13^ and may be struggling with mood disorder and difficulties with problem-solving – problems that can exacerbate difficulties in calling on the assistance of others. The social network can be a positive resource or, on the other hand, a source of the adversity underlying self-harm, and it is not easy to get a clear picture during a single brief encounter: the result is that it can be neglected or misunderstood, especially during an assessment oriented towards the identification of individual pathology. Personal accounts, especially given by those with a history of repeated self-harm, indicate that conversations with staff can emphasise strongly this assumption of individual responsibility, for example by referring pointedly to the person's mental capacity.

The second substantial problem arises from the organisational context within which planning usually takes place. In truth, service provision for many people after self-harm is poor. Typically, self-harm, despite its associated suicide risk, is not seen as falling within the remit of community mental health teams or Improving Access to Psychological Therapies (IAPT) services and yet there are very few specialist clinics. Again, from accounts of people with personal experience, this general lack of provision is exacerbated by the prevalence of the unhelpful concept of non-suicidal self-injury^14^ – a diagnosis that suggests (misleadingly) that the individual is at low risk of suicide and that can therefore make accessing services difficult because the problem is not seen as sufficiently severe.

The worst case, then, is that a clumsily negotiated or unilaterally developed safety plan, coupled with inaccessible professional aftercare, leads to a sense of being left alone in managing the impulse to self-harm and its attendant dangers. We do not know the frequency with which these negative outcomes occur, because the relevant research has not been undertaken.

## Can we mitigate the potential harms of poorly managed safety planning?

One of the recurring complaints about self-harm services is that risk assessment is so often delivered as a thoughtless box-ticking exercise. To avoid safety planning going the same way it has to be delivered as a genuinely collaborative effort. A pre-printed form with little space for personalisation will not convey the right message or serve the purpose. Staff who are going to be undertaking safety planning should therefore be trained in techniques for joint planning – which may be drawn from those with expertise in techniques such as shared decision-making.^15^

Quality improvement projects should be aimed not just at monitoring comprehensiveness of coverage; they could usefully explore the experience of safety planning from the perspective of people who have attended hospital after self-harm – including their level of personal involvement in the content and their sense of the usefulness of the plans. A starter might be the measure developed by NICE.^16^

Making safety planning meaningful depends on the accuracy and usefulness of nominated sources of support, and yet informal sources can be difficult to identify and engage in a single session after an episode of self-harm, while professional sources (such as specialist services) are not available in most places, even at the level of telephone follow-up. If we are to be serious about making self-harm ‘a key area for action’ then we must press for proper professional services for those seen after self-harm – to allow immediate follow-up for help responding to current circumstances and in the longer-term to offer therapeutic support for change. It is these services that will allow resolution of the dilemma of care – making risk reduction meaningful without allowing risk management to define the healthcare response to self-harm.

Research is needed to determine the effectiveness and safety of safety planning, as an adjunct or alternative to standard assessments and follow-up planning. Research in other areas has usefully shown that an important moderator of outcomes is the degree to which there is genuine collaborative engagement of patients with planning,^17^ and such process evaluation would be an important component of any future evaluation.

## About the author

**Allan House** is Emeritus Professor of Liaison Psychiatry in the Leeds Institute of Health Sciences, School of Medicine, University of Leeds, UK. His research interests include the overlap between physical and mental disorder, medically unexplained symptoms, suicide and self-harm.
